# Feasibility Pilot of Personalized Dietary Intervention to Improve Metabolite Profiling and Diabetes Outcomes

**DOI:** 10.1093/geroni/igaf122.4121

**Published:** 2025-12-31

**Authors:** Jisook Ko, Jane Chung, Chun-Liang Chen

**Affiliations:** University of Utah, Salt Lake City, Utah, United States; Emory University, Atlanta, Georgia, United States; UT Health San Antonio, San Antonio, Texas, United States

## Abstract

Effective dietary modification is crucial for managing type 2 diabetes in older adults, as it helps regulate blood glucose and prevent further severe complications. However, varying metabolic responses, cultural food choices, and low adherence present challenges, especially for ethnic minority populations. We conducted a NIH-funded 4-week feasibility pilot of Personalized Behavioral Nutrition (PBN) in 12 older Korean Americans with type 2 diabetes. The PBN program began with a 7-day assessment of diet and fasting blood glucose prior to the intervention, followed by individualized recommendations targeting three specific nutrients delivered by an interventionist. Participants were involved in digital self-monitoring of food intake and fasting glucose using mobile apps and wearable devices during the intervention period. Measures included serum metabolite profiling to assess PBN adherence, lipids panel, fasting glucose, and HbA1C which were collected at baseline and 4 weeks. Analysis of serum samples identified 28 metabolites consistently present at both time points out of 203 measured. Following the intervention, increases in ornithine, D-ribulose-5-phosphate, L-arginine, urea, and 3-methylhistidine were found, suggesting changes in protein metabolism and nitrogen processing, possibly linked to higher B vitamin intake. Increased propionate indicated greater gut microbial fermentation, likely due to higher fiber consumption. Clinically, participants demonstrated a reduction in HbA1C and fasting glucose levels. These findings show that a personalized, culturally informed dietary approach is feasible and may improve metabolic outcomes in older adults with type 2 diabetes. Metabolite profiling can further help identify biomarkers and inform precision nutrition strategies for the geriatric population.

